# Triptolide Inhibits the Proliferation of Prostate Cancer Cells and Down-Regulates SUMO-Specific Protease 1 Expression

**DOI:** 10.1371/journal.pone.0037693

**Published:** 2012-05-30

**Authors:** Weiwei Huang, Tiantian He, Chengsen Chai, Yuan Yang, Yahong Zheng, Pei Zhou, Xiaoxia Qiao, Bin Zhang, Zengzhen Liu, Junru Wang, Changhong Shi, Liping Lei, Kun Gao, Hewei Li, Sue Zhong, Libo Yao, Meng-Er Huang, Ming Lei

**Affiliations:** 1 Key Laboratory of Agricultural Molecular Biology, College of Life Science, Northwest A&F University, Yangling, Shaanxi Province, People's Republic of China; 2 UMR3348 Centre National de la Recherche Scientifique, Institut Curie, Université Paris-Sud 11, Orsay, France; 3 State Key laboratory of Tumor Biology, The Fourth Military Medical University, Xi'an, Shaanxi Province, People's Republic of China; 4 Xi'an San-Yao Bio-pharmaceutical Corporation, Xi'an, Shaanxi Province, People's Republic of China; 5 State Key Laboratory of Applied Organic Chemistry, Lanzhou University, Lanzhou, Gansu Province, People's Republic of China; 6 School of Chinese Materia Medica, Beijing University of Chinese Medicine, Beijing, People's Republic of China; Stanford University, United States of America

## Abstract

Recently, traditional Chinese medicine and medicinal herbs have attracted more attentions worldwide for its anti-tumor efficacy. Celastrol and Triptolide, two active components extracted from the Chinese herb *Tripterygium wilfordii* Hook F (known as Lei Gong Teng or Thunder of God Vine), have shown anti-tumor effects. Celastrol was identified as a natural 26 s proteasome inhibitor which promotes cell apoptosis and inhibits tumor growth. The effect and mechanism of Triptolide on prostate cancer (PCa) is not well studied. Here we demonstrated that Triptolide, more potent than Celastrol, inhibited cell growth and induced cell death in LNCaP and PC-3 cell lines. Triptolide also significantly inhibited the xenografted PC-3 tumor growth in nude mice. Moreover, Triptolide induced PCa cell apoptosis through caspases activation and PARP cleavage. Unbalance between SUMOylation and deSUMOylation was reported to play an important role in PCa progression. SUMO-specific protease 1 (SENP1) was thought to be a potential marker and therapeutical target of PCa. Importantly, we observed that Triptolide down-regulated SENP1 expression in both mRNA and protein levels in dose-dependent and time-dependent manners, resulting in an enhanced cellular SUMOylation in PCa cells. Meanwhile, Triptolide decreased AR and c-Jun expression at similar manners, and suppressed AR and c-Jun transcription activity. Furthermore, knockdown or ectopic SENP1, c-Jun and AR expression in PCa cells inhibited the Triptolide anti-PCa effects. Taken together, our data suggest that Triptolide is a natural compound with potential therapeutic value for PCa. Its anti-tumor activity may be attributed to mechanisms involving down-regulation of SENP1 that restores SUMOylation and deSUMOyaltion balance and negative regulation of AR and c-Jun expression that inhibits the AR and c-Jun mediated transcription in PCa.

## Introduction

The steady increase in the incidence and mortality rates of cancers urges researchers to make great effort on searching for novel anti-tumor drugs or therapies. Extracted compounds from natural herbs, such as Taxol, have been widely used in cancer therapy. Traditional Chinese medicine promises an important and useful alternative in cancer treatment. Many active compounds extracted from Chinese herbs have shown anti-tumor efficacy. Triptolide and Celastrol, two active components extracted from the Chinese herb *Tripterygium wilfordii* Hook F (known as Lei Gong Teng or Thunder of God Vine) used for rheumatoid arthritis therapy, have shown anti-tumor effect and apoptosis induction [1], [2]. Celastrol has been identified as a natural proteasome inhibitor that causes the accumulation of ubiquitinated proteins and proteasome substrates IκB-α, Bax, and p27. Celastrol also induces apoptosis in PCa cells and shrinks the xenografted tumor in mice [1]. Triptolide is a diterpene lactone with potent immunosuppressive effects and anti-tumor properties in different cancers, including melanoma [2], breast cancer [3], pancreatic cancer [4], prostate cancer (PCa) [5] and others. Triptolide induces cell apoptosis via inhibiting HSP70 in pancreatic cancer cells [4], [6], and interrupts the IL6R-JAK/STAT pathway in colon cancer cells [7]. In human anaplastic thyroid carcinoma cells, Triptolide significantly reduces the expression of the NF-kappa B target genes cyclin D1, vascular endothelial growth factor (VEGF), and urokinase-type plasminogen activator [8]. Triptolide acts either independently of or partly dependently on p53 to inhibit solid xenografted tumors growth in mice [9], [10]. However, the efficacy and molecular mechanism of Triptolide on PCa are less studied.

SUMOylation is a novel ubiquitin-like post-translational modification. Four different SUMO proteins, SUMO-1, SUMO-2, SUMO-3 and SUMO-4, have been identified [11]. Similar to ubiquitination, SUMOyaltion involves a series of enzymatic processes. The mature SUMO is activated by conjugation to the E1 enzyme (SAE1/SAE2), transferred to the E2 enzyme (Ubc9) and ligated to the specific lysine residue of the target proteins by an E3 enzyme [11]. SUMOylation modulates multiple cell biological processes such as nuclear transport, cell cycle, chromatin remodeling, transcriptional regulation, DNA repair, and altering proteins ubiquitination and degradation [12]. Ample evidence has shown that SUMOylation is involved in development of human diseases including cancer.

SUMOylation is a reversible process. The conjugated SUMO molecules can be cleaved by SUMO-specific proteases (SENPs). Six SENP proteins have been identified which deSUMOylate target proteins in different ways. SENP1 and SENP2 can remove all 3 SUMOs from target proteins, whereas other SENPs show specificity for SUMO-2 and SUMO-3 [13]. DeSUMOylation has been demonstrated to involve in the human diseases progression [14] such as PCa [15] and breast cancer [16]. Previously, Cheng et al [15] reported that deSUMOylation plays an important role in the development of PCa. They found that SENP1 was over-expressed in human PCa specimens but not in normal human prostate cells. siRNA inhibition of SENP1 reduced PCa cells growth. In addition, their initial results *in vivo* in transgenic mice indicated that over-expression of SENP1 leads to the development of prostatic intraepithelial neoplasia (PIN) at an early age. Further, SENP1 markedly enhances the activity of AR–dependent transcription by deSUMOylation of HDAC1 [17] and c-Jun–dependent transcription by deSUMOylating the CRD1 domain of p300 [18], which all involved in the tumorigenesis of PCa. Overall, these data suggest SENP1 play an important role in PCa progression and could be a novel PCa marker and therapy target.

In the present study, we demonstrated that Triptolide significantly inhibited PCa cell proliferation *in vitro* and xenografted PC-3 tumor progression *in vivo*, Triptolide also induced apoptosis in PCa cells. The anti-tumor efficacy of Triptolide was more potent than Celastrol. Meanwhile, we found Triptolide significantly down-regulated the SENP1 expression in both mRNA and protein levels, resulting in an enhanced cellular SUMOylation in PCa cells. Furthermore, Triptolide also negatively regulated AR and c-Jun expression and suppressed AR and c-Jun transcription. Overall, our experimental results suggest that Triptolide is a natural potential compound for PCa therapy.

## Results

### Triptolide significantly inhibited PCa cell proliferation *in vitro*

**Figure 1:**
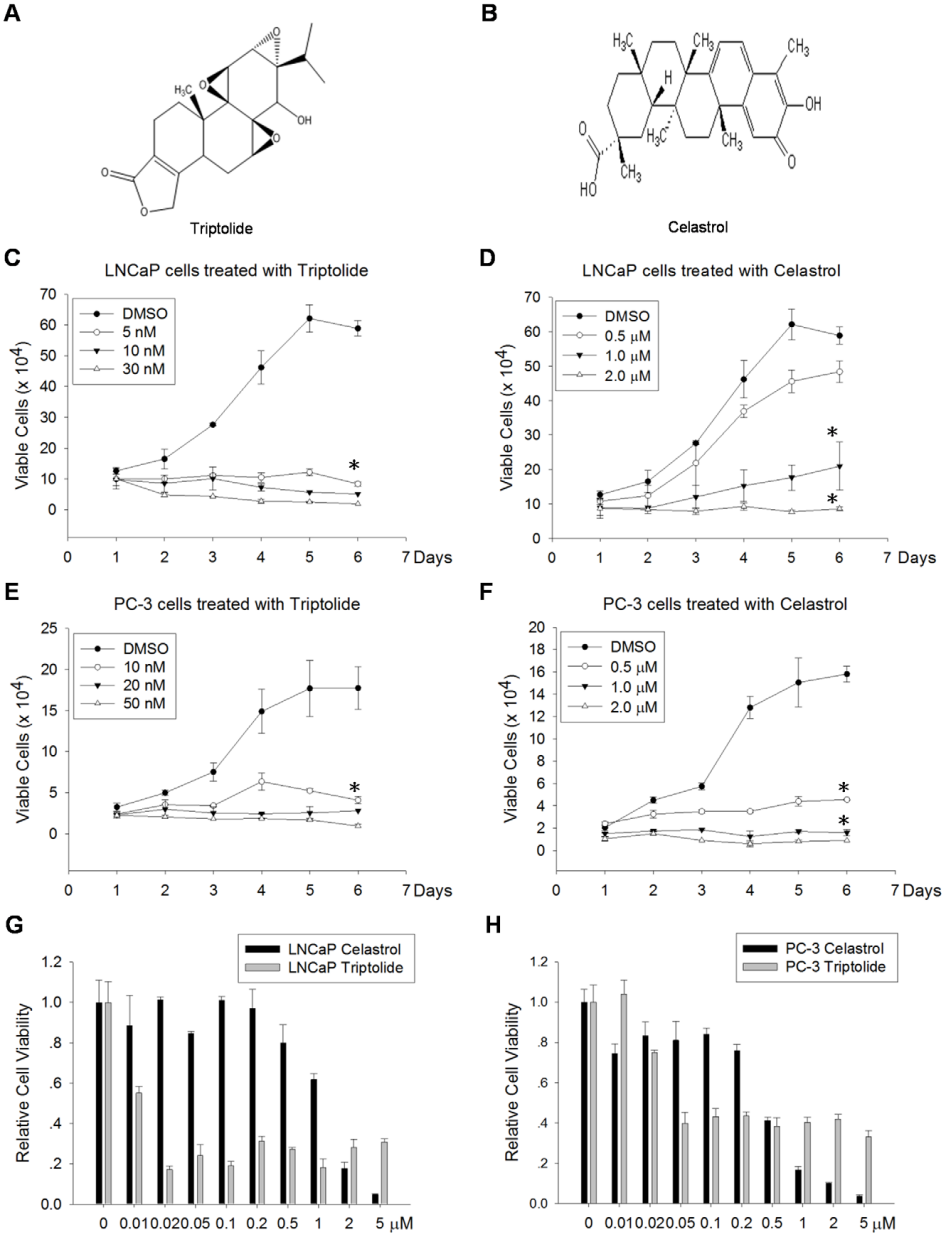
Triptolide and Celastrol inhibited cell growth and induced cell death in PCa cell lines. (A) and (B) The chemical structure of Triptolide (A) and Celastrol (B), respectively. (C), (D), (E) and (F) PCa cells were treated with indicated doses of Triptolide or Celastrol, viable cell numbers were counted every day for 6 days. All assays were performed in triplicate, and data shown are mean ± SD of three independent experiments. * indicates P<0.05 (compared to control). (C) Effect of Triptolide on LNCaP cells. (D) Effect of Celastrol on LNCaP cells. (E) Effect of Triptolide on PC-3 cells. (F) Effect of Celastrol on PC-3 cells. (G) and (H) PCa Cells were treated with indicated concentrations of Triptolide or Celastrol and cells viability was determined by MTT assay. All assays were performed in triplicate, and data shown are mean ± SD of four independent experiments. (G) Effect of Triptolide and Celastrol on LNCaP cells. (H) Effect of Triptolide and Celastrol on PC-3 cells.

Both Triptolide (Fig. 1A) and Celastrol (Fig. 1B) belong to terpenes and are two active components extracted from the Chinese herb *Tripterygium wilfordii* Hook F [1], [19]. Celastrol, as a potent proteasome inhibitor, inhibits PCa cell proliferation and induces cell apoptosis [1]. Triptolide has been reported to suppress cell proliferation in many types of cancer, but its efficacy on PCa and target molecules have not been clearly studied. To determine the anti-tumor activity of Triptolide on PCa cells and compare with Celastrol, we performed growth curve assay and cell viability assay using two PCa cell lines, LNCaP (androgen-dependent) and PC-3 cells (androgen-independent). We confirmed that both Triptolide and Celastrol caused significant inhibition of proliferation in LNCaP and PC-3 cells. In the growth curve assay, Triptolide significantly inhibited the proliferation of PCa cells even at doses of 5 nM in LNCaP cells (Fig. 1C) and 10 nM in PC-3 cells (Fig. 1E). In contrast, Celastrol only suppressed PCa cells growth at high doses such as 1 µM in LNCaP cells (Fig. 1D) and 0.5 µM in PC-3 cell (Fig. 1F). These results showed that Triptolide can efficiently suppress PCa cell proliferation. To further determine the cytotoxic effect of Triptolide on PCa cell and compare with Celastrol, we carried out cell viability assay using MTT method. Both PCa cell lines were treated with broad doses of Triptolide or Celastrol, from 0.01 µM to 5 µM, for 48 h. As shown in Figure 1G, at low dose (such as 0.02 µM), Triptolide was sufficient to kill majority LNCaP cells but residual proportion of viable cells persisted even in the presence of higher concentrations of Triptolide. It was in contrast to the dose-dependent cytotoxic effect of Celastrol. However, a much higher dose of Celastrol was required to attain a similar cytotoxic effect with 0.02 µM Triptolide. Similar pattern was observed in cell viability assay for PC-3 cells (Fig. 1H). To quantify the efficacy of two compounds on PCa cell, we calculated the IC_50_ value according to the cell viability data. Triptolide had a much lower IC_50_ values for both cell lines compared to Celastrol (Table 1). These data demonstrated that Triptolide is an effective agent to inhibit PCa cell proliferation and to induce cell death.

**Table 1:** IC_50_ values.

Name	Time (h)	IC50 (µM)	Cell
Celastrol	48	0.4881	PC-3
Triptolide	48	0.0203	PC-3
Celastrol	48	1.195	LNCaP
Triptolide	48	0.009754	LNCaP

### Triptolide induced apoptosis in PCa cells *in vitro*

**Figure 2:**
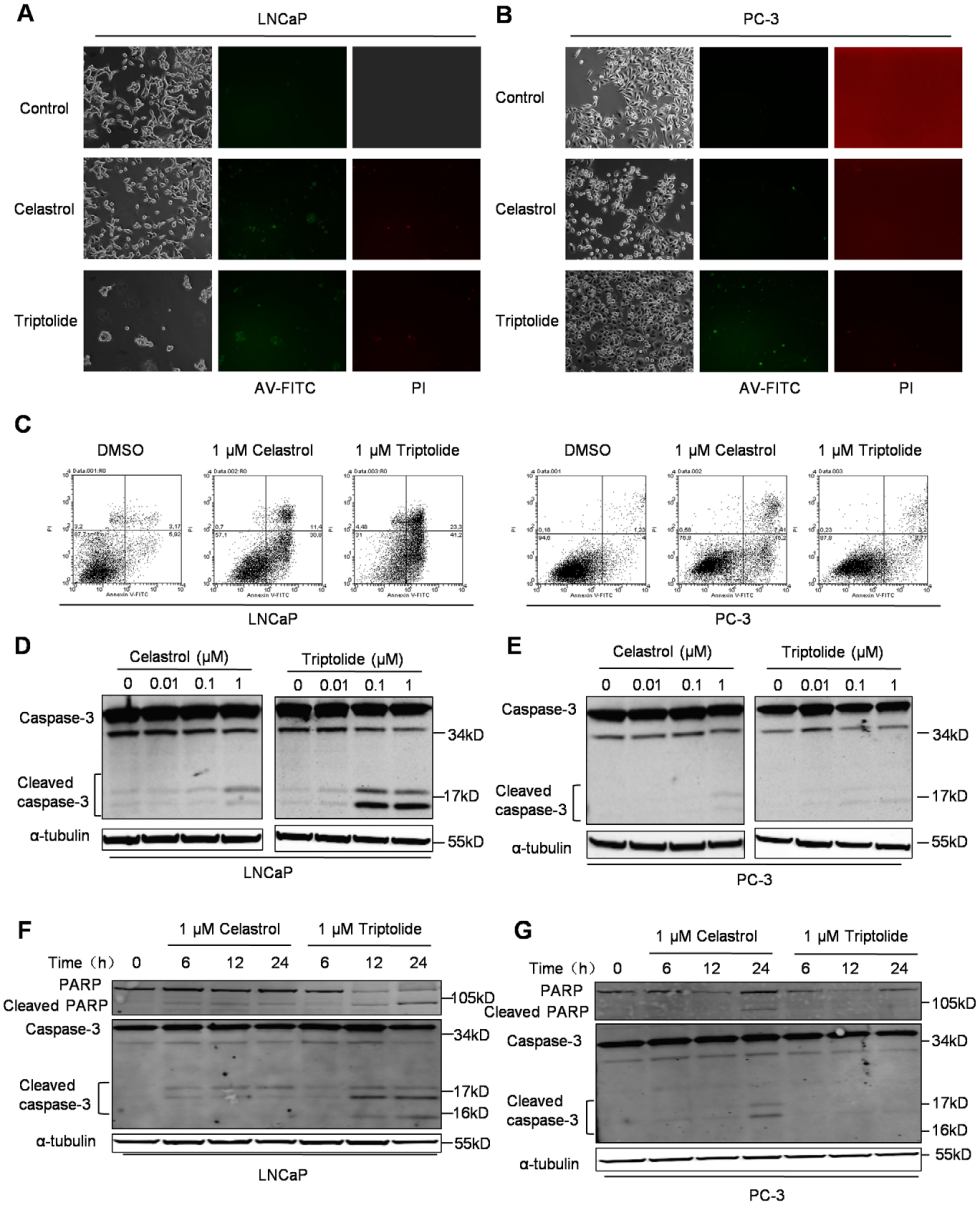
Triptolide and Celastrol induce apoptosis in PCa cells *in vitro*. (A) and (B) Fluorescence microscopy observation of Triptolide- and Celastrol- induced apoptosis in LNCaP (A) and PC-3 (B) cells. After treatment with 1 µM Celastrol or Triptolide for 24 h, LNCaP and PC-3 cells were incubated with AV-FITC (green) and PI (red). Representative bright field and fluorescent images are shown. (C) Flow cytometric detection of apoptosis in LNCaP and PC-3 cells treated as above. Percentage of intact cells (AV−/PI−), early apoptotic cells (AV+/PI−), late apoptotic/necrotic cells (AV+/PI+) and necrotic cells (AV−/PI+) are presented. (D) and (E) Western blot analysis of caspase-3 protein in Triptolide- or Celastrol-treated LNCaP (D) and PC-3 (E) cells. Cells were treated with indicated concentrations of Triptolide or Celastrol for 24 h and subjected to analysis. Uncleaved caspase-3 and cleaved products are indicated. α-tubulin was used as a loading control. (F) and (G) Western blot analysis of PARP and caspase-3 proteins in Triptolide- or Celastrol-treated LNCaP (F) and PC-3 (G) cells. Cells were treated with 1 µM Triptolide or Celastrol for desired times. Uncleaved PARP and caspase-3 and their cleaved products are indicated. α-tubulin was used as a loading control.

We next assessed whether Triptolide- and Celastrol-induced cell death corresponded to an apoptotic response. LNCaP and PC-3 cells were treated with 1 µM Triptolide and Celastrol for 24 h, respectively, stained with fluorescein isothiocyanate (FITC)-conjugated annexin V (AV) and 50 µg/ml propidium iodide (PI), then analyzed by fluorescence microscopy and by flow cytometry. The simultaneous detection of phosphatidylserine externalization and loss of membrane integrity with AV-FITC and PI, respectively, discriminates between early apoptosis (AV+), late apoptosis eventually leading to secondary necrosis (AV+/PI+) and primary necrosis (PI+). As shown in Figure 2A and Figure 2B, treatment with Triptolide and Celastrol resulted in a higher proportion of cells with positive AV and/or PI staining. Flow cytometric analysis showed that treatment with Triptolide and Celastrol caused respectively 40% and 30% of LNCaP cells to be AV+/PI−, whereas only <6% of vehicle (DMSO)-treated cells showed an apoptotic response. The two compounds had relatively moderate effect in inducing apoptosis in PC-3 cells, 8.77% and 15.2% respectively, versus 4% in vehicle (DMSO)-treated cells (Fig. 2C and Fig. S1). These results showed that both Triptolide and Celastrol induced apoptosis in PCa cells. In addition, LNCaP cells were more sensitive to Triptolide and Celastrol than PC-3 cells. It has been reported that Triptolide induces apoptosis through caspase cascade [4]–[6]. To further determine whether caspase is involved in Triptolide-induced apoptosis in PCa cells, we investigated the status of caspase-3 in PCa cells treated with various doses Triptolide or Celastrol for 24 h or with 1 µM Triptolide and Celastrol for desired times. Activation of caspases-3 was monitored by Western blot using a polyclonal antibody that recognize both procaspase-3 and its active cleavage fragments, p17 and p12. As shown in Figure 2D–2E, the cleavage of procaspase-3 was enhanced in Triptolide-treated LNCaP cells, and to a less degree, in Celastrol-treated LNCaP cells. Only very low levels of caspase-3 fragments were detectable in Celastrol or Triptolide-treated PC-3 cells. We then followed the status of nuclear enzyme poly (ADP-ribose) polymerase (PARP) that is one of the main cleavage targets of Caspase-3. PARP cleavage was enhanced in Triptolide-treated LNCaP cells, and to a less degree, in Celastrol-treated LNCaP cells (Fig. 2F). Again, lower levels of PARP fragments were detectable in Celastrol or Triptolide-treated PC-3 cells (Fig. 2G). Altogether, these data demonstrated that Triptolide, and to a less degree, Celastrol, induce apoptosis in PCa cells associated with activation of caspase-3 and cleavage of PARP.

### Triptolide suppressed xenografted PC-3 tumor growth *in vivo*

**Figure 3:**
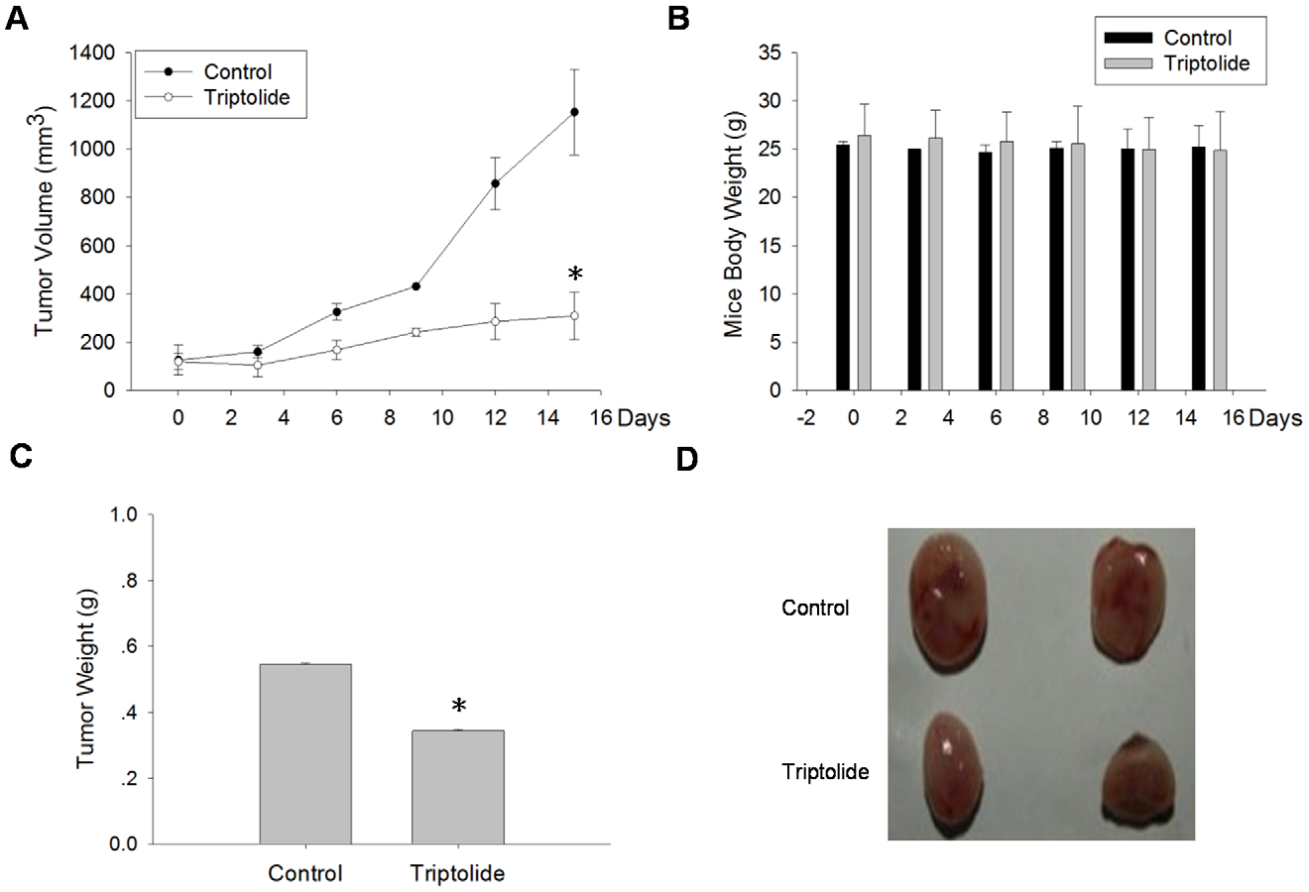
Triptolide suppressed PC-3 tumor progression in mouse xenograft model. PC-3 cells (2×10^6^ per mouse) were injected into 5-week-old nude mice. When solid tumors grew to about 100 mm^3^, the mice were treated intraperitonneally with either a vehicle control or Triptolide at 0.4 mg/kg daily for 15 days. Tumor sizes and body weight were measured every three days. (A) Effect of Triptolide on xenograft tumor volume. (B) Effect of Triptolide on nude mice body weight. (C) Effect of Triptolide on xenograft tumor weight. (D) Image of xenograft tumor treated with or without Triptolide.* indicates P<0.05 (compared to control).

We observed that Triptolide can inhibit PCa cell proliferation and induce cell apoptosis *in vitro*. To determine the efficacy of Triptolide *in vivo*, we carried out xenograft study. PC-3 cells were implanted subcutaneously in nude mice. When the tumors became palpable (∼100 mm^3^), mice were treated intraperitonneally with either a vehicle control or Triptolide at 0.4 mg/kg daily for 15 days. Tumor sizes and body weight were measured every three days. As shown in Figure 3, the volume (Fig. 3A) and weight (Fig. 3C) of xenograft tumors treated with Triptolide were significantly smaller than those in the control group (Fig. 3D), indicating that Triptolide has potent anti-tumor effect *in vivo*. Meanwhile, there was no significant difference in body weight between the treated and the control group (Fig. 3B), suggesting that administered dose of Triptolide has no significant toxicity to mice.

### Triptolide down-regulated SENP1 expression in PCa cells

Since Triptolide inhibited PCa cell proliferation and induced cells apoptosis *in vitro*, and suppressed xenografted tumor growth *in vivo*, we were interested in the mechanisms underlying these anti-tumor effects. Previous studies showed that SENP1 is over-expressed in PCa cells and tumors samples [15]. SENP1 over-expression in transgenic mice promotes the development of malignant lesions in the prostate gland [15]. These data suggest that over-expression of SENP1 mediates PCa development and SENP1 could be a potential target for PCa therapy.

**Figure 4:**
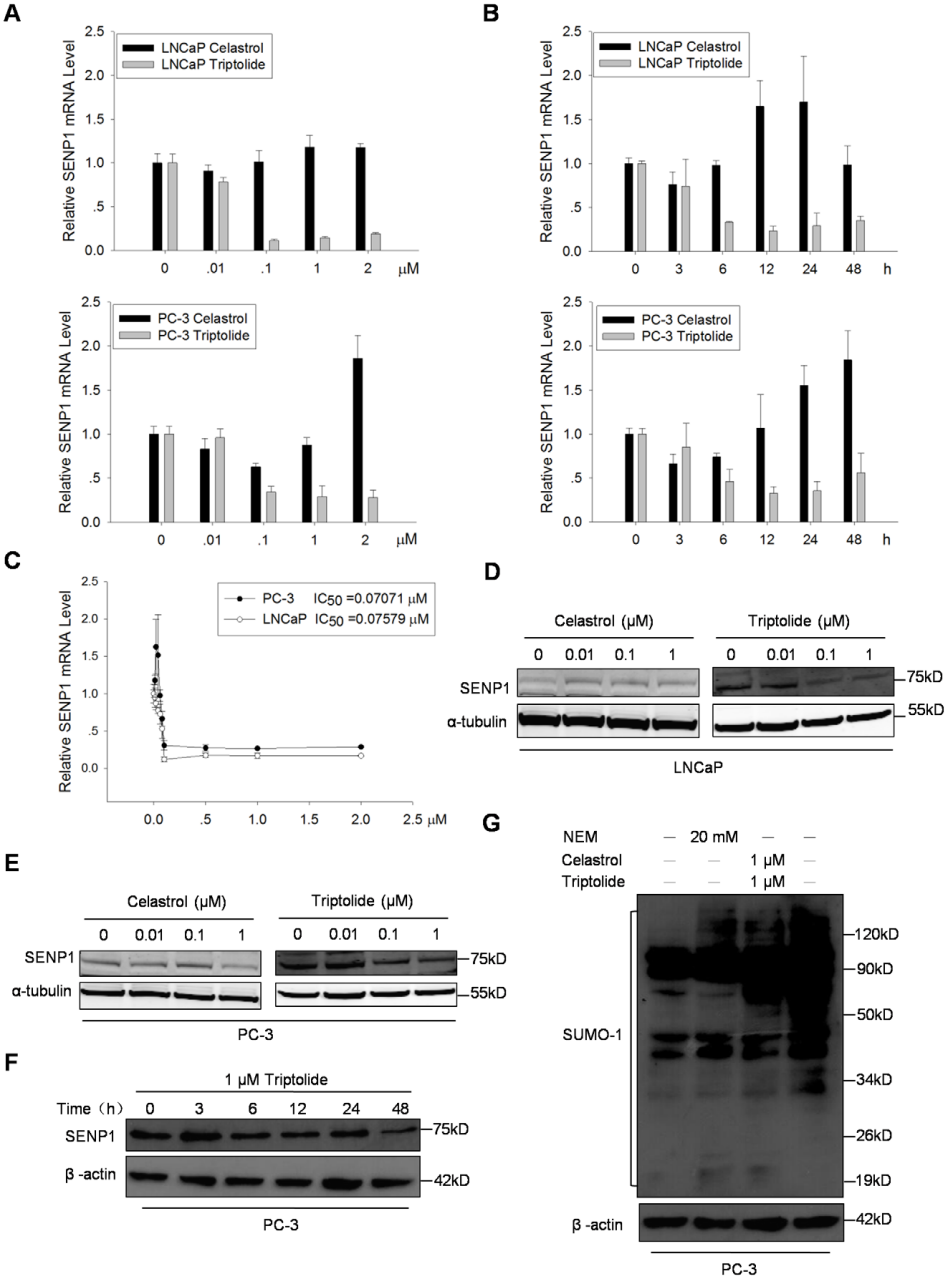
Triptolide down-regulated SENP1 expression in PCa cells. (A) Triptolide decreased SENP1 mRNA level in both LNCaP and PC-3 cells in a dose-dependent manner. Cells were treated with indicated doses of Triptolide or Celastrol for 24 h before analysis. qRT-PCR were performed using the specific primers for SENP1 and β-actin (used as an internal control). (B) Triptolide decreased SENP1 mRNA level in both LNCaP and PC-3 cells in a time-dependent manner. Cells were treated with 0.1 µM Triptolide or Celastrol for indicated times, SENP1 and β-actin mRNA levels were determined by qRT-PCR. (C) Effect of Triptolide on SENP1 mRNA level in PCa cells. IC_50_ was shown as calculated by Prism 5.04. (D) and (E) Triptolide decreased SENP1 protein level in LNCaP (D) and PC-3 (E) in a dose-dependent manner. Cells were treated with indicated doses of Triptolide or Celastrol for 24 h before Western blot analysis. α-tubulin was used as a loading control. (F) Triptolide decreased SENP1 protein level in PCa cells in a time-dependent manner. PC-3 cells were treated with 1 µM Triptolide for indicated time before Western blot analysis. β-actin was used as a loading control. (G) Triptolide enhanced cellular SUMOylation in PC-3 cells. PC-3 cells were treated with 1 µM Triptolide or Celastrol for 24 h before Western blot analysis using SUMO-1 antibody. NEM was used as a positive control for a SUMO protease inhibitor.

To investigate whether Triptolide regulates SENP1 expression, firstly we analyzed SENP1 mRNA level by real-time PCR in LNCaP and PC-3 cells treated with various doses of Triptolide or Celastrol for 24 h, or treated with 0.1 µM Triptolide or Celastrol for desired times. As shown in Figure 4A and 4B, Triptolide significantly decreased SENP1 mRNA levels in both LNCaP and PC-3 cells in dose-dependent and time-dependent manners. Meanwhile, Celastrol also reduced SENP1 mRNA level following a brief treatment at low doses. In contrast, at high doses and with longer treatment time, Celastrol enhanced SENP1 mRNA level in both treated PCa cell lines. To evaluate the efficacy of Triptolide-induced down-regulation of SENP1 expression, we determined the dose of Triptolide required to induce 50% decrease in SENP1 mRNA (IC_50_). Both LNCaP and PC-3 cells were treated with more than 6 doses Triptolide for 24 h, as shown in Figure 4C, Triptolide efficiently induced the decrease in SENP1 mRNA level with IC_50_ value 0.07579 µM and 0.07071 µM for LNCaP and PC-3 cells respectively. We further measured the SENP1 protein level in PCa cells treated with Triptolide or Celastrol. Triptolide was able to decrease the SENP1 protein level in both dose-dependent (Fig. 4D, 4E) and time-dependent (Fig. 4F) manners. In contrast, Celastrol did not significantly reduce SENP1 protein level in treated PCa cells. These results are consistent with our qRT-PCR results and indicate that Triptolide suppresses SENP1 expression in PCa cells at both mRNA and protein levels.

SENP1 plays an important role in SUMOylation processes. As an isopeptidase, SENP1 deconjugates SUMO from SUMOylated protein. A reduced SENP1 expression in Triptolide-treated PCa cells would result in an enhanced cellular SUMOylation. To verify this expectation, the cellular SUMOylation level in PC-3 cells treated with 1 µM Triptolide or Celastrol for 24 h was detected by Western blot using SUMO-1 antibody. Cells without treatment but lysed in lysis buffer in the presence of 20 mM N-ethylmaleimide (NEM), a potent inhibitor of deubiquitinating enzymes and of SUMO hydrolase activity, was used a positive control [20]. As shown in Figure 4G, compare to control, Triptolide treatment significantly enhanced total SUMOylation level. Interestingly, Celastrol also enhanced total SUMOylation level, but with less efficiency compared to Triptolide, which could result from its proteasome suppression activity. It was reported that SUMOylation of some proteins, such as HIF1 [12] and PML [21], promotes these proteins ubiquitin-dependent degradation. As a proteasome inhibitor, Celastrol could suppress degradation of these SUMOylated proteins and induce their accumulation. To confirm this result, we performed similar experiments using another SUMO-1 monoclonal antibody. The western blot results showed that the levels of SUMO-1 heterodimers were increased in Triptolide treated samples (Fig. S2A, S2B). We also found that the level of SUMO-1 monomer was reduced in Triptolide treated samples (Fig. S2C). This could result from either down-regulated SENP1 expression, or the increase of SUMO-1 heterodimers. Overall, Triptolide is able to negatively regulate SENP1 expression at both mRNA and protein levels, resulting in an enhanced cellular SUMOylation level in PCa cells.

### Triptolide suppressed androgen receptor (AR) expression in LNCaP cells

**Figure 5:**
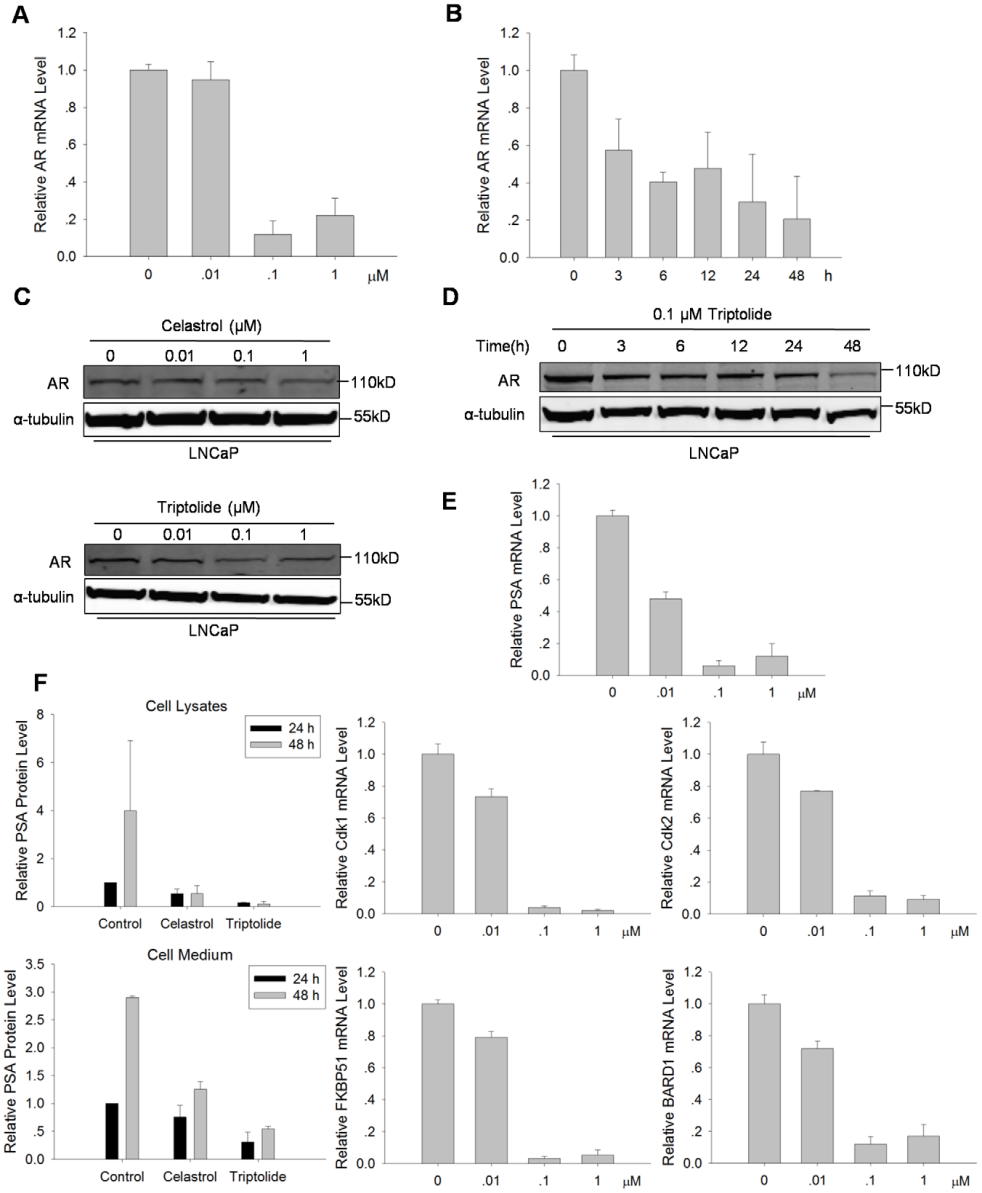
Triptolide down-regulated AR expression in LNCaP cells. (A) Triptolide decreased AR mRNA level in LNCaP cells in a dose-dependent manner. LNCaP cells were treated with indicated doses of Triptolide for 24 h before analysis. qRT-PCR was performed using the specific primers for AR and β-actin. (B) Triptolide decreased AR mRNA level in LNCaP cells in a time-dependent manner. LNCaP cells were treated with 0.1 µM Triptolide for indicated times before analysis. (C) Triptolide and Celastrol decreased AR protein level in LNCaP cells in a dose-dependent manner. LNCaP cells were treated with indicated doses of Triptolide or Celastrol for 24 h before Western blot analysis. α-tubulin was used as a loading control. (D) Triptolide decreased AR protein level in LNCaP cells in a time-dependent manner. LNCaP cells were treated with 0.1 µM Triptolide for indicated times before Western blot analysis. α-tubulin was used as a loading control. (E) Triptolide decreased AR target genes expression in LNCaP cells. LNCaP cells were treated with indicated doses of Triptolide for 24 h before qRT-PCR analysis using respective specific primers. (F) Triptolide inhibited PSA protein level in LNCaP cells. LNCaP cells were treated with Triptolide or Celastrol for 24 h or 48 h. Aliquots of medium and cell lysis from the treated cells were collected and the total PSA level was measured by CLIA.

AR plays an important role in normal prostate development and maintenance, and in prostate cancer progression. Celastrol was reported to suppress AR expression in LNCaP cells [1]. We raised the question whether Triptolide inhibits AR expression and AR-mediated transcription in PCa cells. We analyzed AR mRNA level in LNCaP cells treated with various doses of Triptolide and Celastrol for 24 h or with 0.1 µM Triptolide and Celastrol for desired times by real-time PCR. As shown in Figure 5A and 5B, Triptolide significantly reduced AR mRNA level in dose-dependent and time-dependent manners. We further analyzed the AR protein level in LNCaP cell treated with Triptolide, with Celastrol-treated cells as positive control. As shown in Figure 5C and 5D, Triptolide reduced AR protein expression in the same manners as mRNA level. Since AR functions through binding to androgen-response-elements (AREs) in the promoter region of target genes and regulates these androgen response genes transcription to induce cell proliferation [22], Triptolide-induced suppression of AR expression could inhibit AR-mediated transcription. To verify this hypothesis, expression of several AR targets genes in AR-positive LNCaP cells treated with various doses of Triptolide for 24 h was detected by qRT-PCR (Fig. 5E). The expression of PSA, BARD1, Cdk1, Cdk2 and FKBP51, known to be positively regulated by AR, were indeed suppressed. These data suggest that Triptolide inhibited AR transcription activity by suppressing AR expression. In addition to the AR target genes, prostate-specific antigen (PSA) is an important biological marker for the clinical diagnosis of human prostate cancer [23], [24]. Therefore, we further detected PSA protein level in LNCaP cell lysate and culture medium by chemiluminescence immunoassay (CLIA). PSA protein level in LNCaP cells and that secreted into medium were decreased following 1 µM Triptolide treatment (Fig. 5F), while the same concentration of Celastrol showed less suppressive effect on PSA. Overall, these results indicate that Triptolide suppresses AR expression and induces AR-mediated transcription inhibition.

### Triptolide down-regulated c-Jun expression in PCa cells

**Figure 6:**
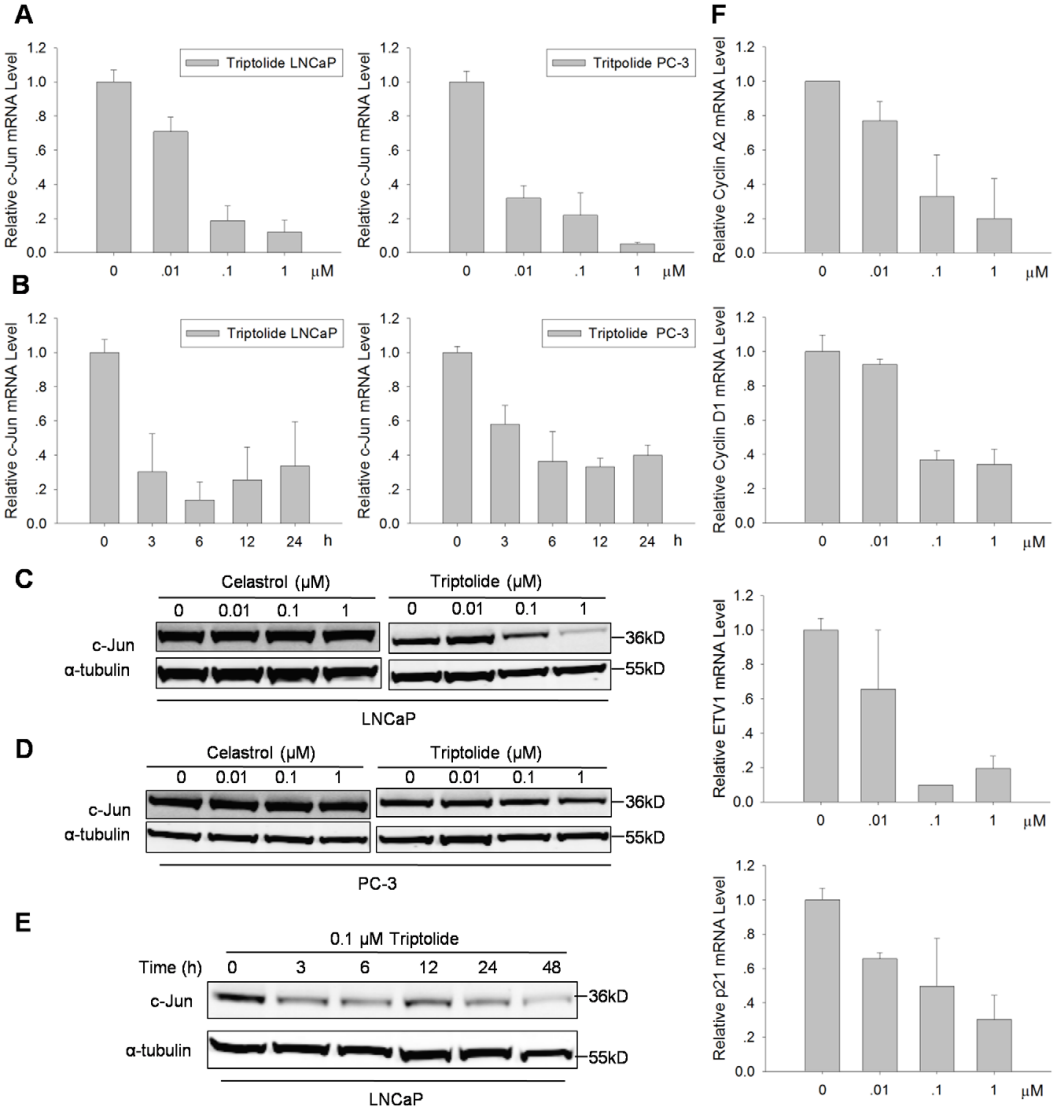
Triptolide down-regulated c-Jun expression in PCa cells. (A) Triptolide decreased c-Jun mRNA level in LNCaP and PC-3 cells in a dose-dependent manner. Cells were treated with indicated dose of Triptolide for 24 h before RNA extraction and qRT-PCR using the specific primers for c-Jun and β-actin. (B) Triptolide decreased c-Jun mRNA level in LNCaP and PC-3 cells in a time-dependent manner. Cells were treated with 0.1 µM Triptolide for indicated time before RNA extraction and qRT-PCR. (C) and (D) Triptolide decreased c-Jun protein level in LNCaP (C) and PC-3 (D) cells in a dose-dependent manner. Cells were treated with indicated dose of Triptolide or Celastrol for 24 h before Western blot analysis. α-tubulin was used as a loading control. (E) Triptolide decreased c-Jun protein level in LNCaP cells in a time-dependent manner. LNCaP cells were treated with 0.1 µM Triptolide for indicated time before Western blot analysis. α-tubulin was used as a loading control. (F) Triptolide inhibited c-Jun target genes expression in LNCaP cells. LNCaP cells were treated with indicated concentration of Triptolide for 24 h before qRT-PCR analysis of c-Jun target genes using respective specific primers.

Oncoprotein c-Jun which is often over-expressed in cancer cells is involved in PCa transformation [25]. c-Jun is a major component of the transcription factor AP-1 [26], [27]. In addition, c-Jun co-activates AR transcriptional activities, and SENP1 suppresses c-Jun expression [18]. In order to determine whether Triptolide regulates c-Jun expression, we treated PCa cells with various doses Triptolide or Celastrol for 24 h, or with 0.1 µM Triptolide or Celastrol for desired times. As shown in Figure 6A–6D, c-Jun expression, at mRNA and protein levels, was suppressed by Triptolide in dose-dependent and time-dependent manners. In contrast, Celastrol had no effect on c-Jun expression. To further study the effect of Triptolide on activity of c-Jun, we analyzed the c-Jun target genes expression by qRT-PCR. c-Jun target genes Cyclin A2, Cyclin D1, ETV1 and p21, which are known to be up-regulated by c-Jun were suppressed following Triptolide treatment (Fig. 6F). These data indicate that Triptolide suppresses c-Jun expression at mRNA and protein level and resultes in c-Jun mediated transcription inhibition.

### Down-regulation or over-expression of SENP1, c-Jun or AR inhibited Triptolide anti-PCa efficacy

**Figure 7:**
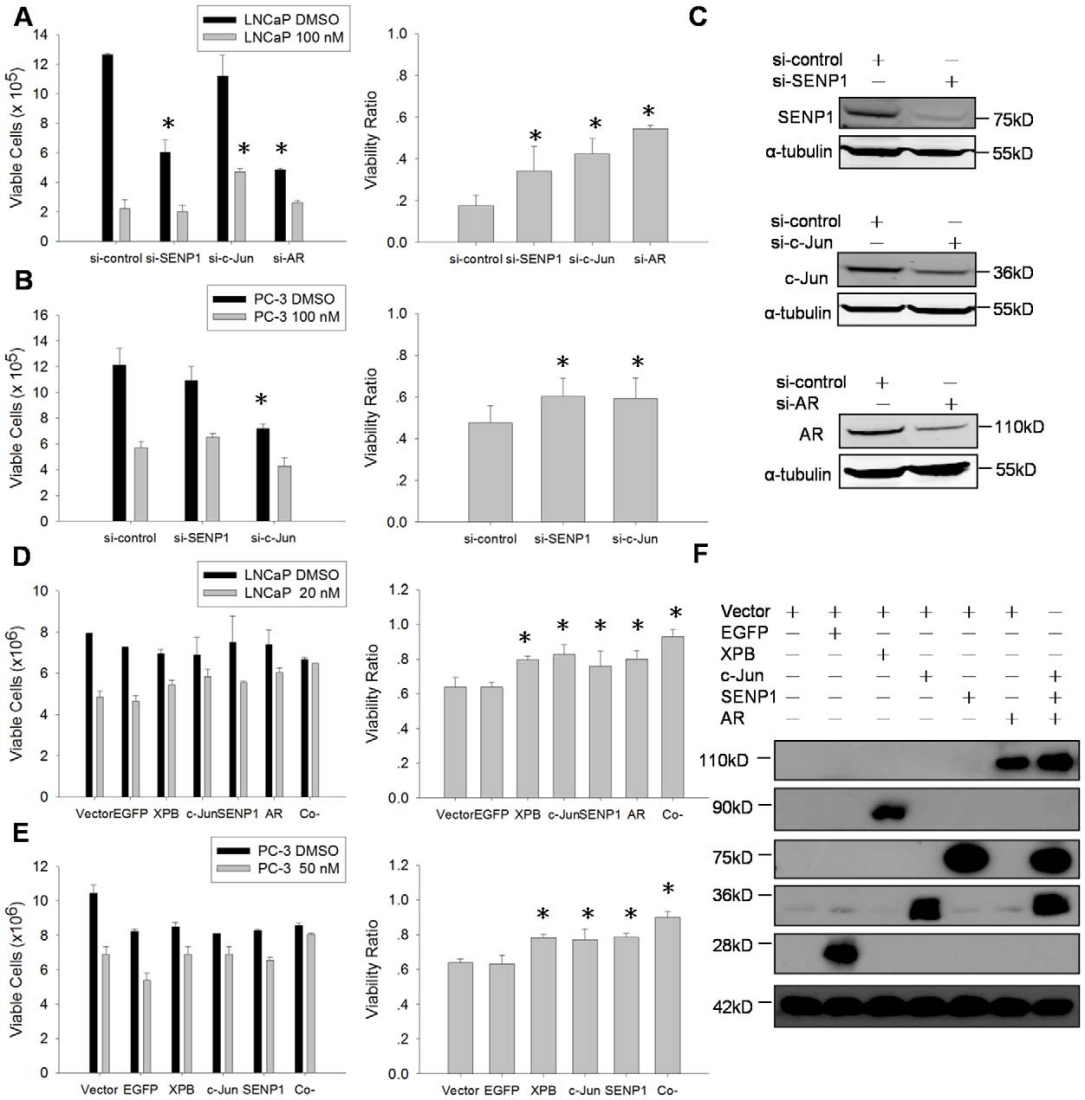
Down-regulation or over-expression of SENP1, c-Jun or AR inhibited Triptolide anti-PCa efficacy. (A) and (B) Down-regulation of SENP1, c-Jun or AR expression increased cell viability upon Triptolide treatment. LNCaP and PC-3 cells were transfected with specific siRNA against SENP1, c-Jun and AR. 24 h after transfection, the transfected cells were treated with 100 nM triptolide or vehicle control for 48 h, then the viable cell number was counted in each treatment. (A) Effect of knockdown SENP1, c-Jun and AR on Triptolide efficacy in LNCaP cell. Left chart shows the viable cell number in each treatment. Right chart shows the viability ratio. (B) Effect of knockdown SENP1 and c-Jun on Triptolide efficacy in PC-3 cell. * indicates P<0.05 (compared to control). (C) Down-regulation of SENP1, c-Jun and AR protein levels by siRNA in (A) and (B). (D) and (E) Over-expression of SENP1, c-Jun and AR rescued cell viability upon Triptolide treatment. PCa cells were transfected or cotransfected with 800 ng SENP1, c-Jun and AR expression plasmids DNA as indicated. EGFP protein was used as a negative control and the Triptolide binding protein XPB was used as a positive control. Empty vector was used as blank control and to keep the total amount of plasmids DNA equal in each well. 48 h after transfection, cells were treated with Triptolide for another 48 h and viable cell number were counted. (D) Effect of ectopic expression or coexpression of SENP1, c-Jun and AR on Triptolide efficacy in LNCaP cells. (E) Effect of ectopic expression or coexpression of SENP1 and c-Jun on Triptolide efficacy in PC-3 cells. * indicates P<0.05 (compared to control). (F) Ectopic expression or coexpression of SENP1, c-Jun and AR in PCa cells evaluated by Western blot using anti-Flag and anti-HA antibodies.

Since we observed that Triptolide suppressed SENP1 expression, enhanced cellular SUMOylation, inhibited AR and c-Jun expression and suppressed AR/c-Jun-mediated transcription, we asked whether Triptolide anti-PCa efficacy relies on SENP1, c-Jun or AR expression in LNCaP and PC-3 cells. To answer this question, firstly we knocked down SENP1, c-Jun or AR expression by specific siRNA in LNCaP (with siRNA of SENP1, c-Jun or AR) and PC-3 (with siRNA of SENP1 or c-Jun) cells. Twenty-four hours after siRNA transfection, cells were treated with 100 nM Triptolide or vehicle control for 48 h, the viable cells number was counted then. Efficiency of siRNA againt SENP1, c-Jun and AR were evaluated by Western blot (Fig. 7C). Interestingly, in the absence of Triptolide treatment, silencing of SENP1, c-Jun or AR in LNCaP cells reduced cellular viability (Fig. 7A). Similarly, silencing of SENP1 or c-Jun in PC-3 cells also decreased cellular viability (Fig. 7B). This observation may indicate that cytotoxicity of Triptolide in PCa cells could result from Triptolide-induced downregulation of SENP1, AR or c-Jun. In order to reveal the effect of Triptolide on the SENP1, AR or c-Jun silenced cells, we introduced the value of ‘the viability ratio’ which is obtained by dividing the viable cell number of the Triptolide-treated group by that of the control group without Triptolide-treatment. Interestingly, knockdown of SENP1, c-Jun or AR appeared to increase cells viability ratio under Triptolide treatment (Fig. 7A, 7B), suggesting higher Triptolide resistance. We further checked the effect of SENP1, c-Jun or AR over-expression on Triptolide anti-PCa efficacy. PCa cells were transfected or co-transfected with SENP1, c-Jun and AR expression plasmids (Fig. 7F). An irrelevant protein EGFP was used as a negative control and a Triptolide binding protein XPB was used as a positive control. 48 h after transfection, cells were treated with Triptolide for another 48 h and viable cells number was counted. Ectopic expression of these proteins were evaluated by Western blot (Fig. 7F). As shown in Fig. 7D–7E, ectopic expression of SENP1, c-Jun or AR significantly increased PCa cells viability ratio under Triptolide treatment, indicating that rescuing these three Triptolie down-regulating proteins expression could inhibit Triptolide cell toxicity. Furthermore, co-expression of these proteins conferred higher cells viability ratio than respective individual expression, suggesting that all these proteins are involved in Triptolide cytotoxity. In contrast, over-expression of an irrelevant protein EGFP did not affect the toxicity of Triptolide, which showed similar cells viability ratio with empty vector control, suggesting that the effect of SENP1, c-Jun or AR over-expression on Triptolide toxicity were indeed resulted from their molecular function. Interestingly, ectopic expression of SENP1, c-Jun or AR showed similar effect under Triptolide treatment as XPB over-expression did. It was reported that Triptolide binds XPB to induce RNA polymerase II–mediated transcription inhibition, suggesting that down-regulation of SENP1, c-Jun and AR expression are important for Triptolide effect. We noticed that ectopic expression of these proteins including EGFP mildly decreased cells number, especially in PC-3 cell line. This may be due to the robust expression of these proteins affect other protein expression. Nevertheless, this effect did not influence Triptolide activity since ectopic expression of EGFP showed similar cell viability ratio with vector control under Triptolide treatment, and ectopic expression or co-expression of SENP1, c-Jun or AR induced cell number increase in Triptolide treatment groups in LNCaP cells comparing with vector control. Taken together, down-regulation or over-expression of SENP1, c-Jun or AR appears to significantly inhibit Triptolide anti-PCa toxicity, indicating that down-regulation of SENP1, c-Jun or AR by Tritpolide is important for Tritplode activity, suggesting these proteins are key targets of Triptolide for its' anti-PCa effect.

## Discussion

The Chinese herb *Tripterygium wilfordii* Hook F has been used for centuries in traditional Chinese medicine for treating fever, chills, edema and carbuncle. Recently, this herb has attracted intensive attention from researcher worldwide for its potent efficacy on many diseases. More than 100 small active compounds have been extracted from this herb. The diterpenoid epoxide Triptolide and the quinine triterpene Celastrol, two main bioactive components of *Tripterygium wilfordii* Hook F, exhibit anti-tumor activity. They have been shown to suppress cell proliferation and induce apoptosis in many types of cancer including breast cancer [3], [28], pancreas cancer [4], [29], prostate cancer [1], [5], [30], either in cell culture assay or in xenografted tumor assay. Celastrol has been identified as a natural 26 s proteasomal inhibitor that has great potential for PCa therapy [1]. But the efficacy of Triptolide on human PCa has been less studied. In this study, we demonstrated that Triptolide suppressed proliferation and induced apoptosis in two PCa cell lines: the androgen-dependent LNCap cells and androgen-independent PC-3 cells. This is in consistent with the findings of Li et al [31]. Furthermore, Triptolide suppressed xenografted PC-3 tumor progression in nude mice. Based on cell viability assay and IC_50_ value, Triptolide exhibited more potent effect than Celastrol on PCa cells and had antiproliferation activity at a nanomolar grade. It was reported that Triptolide inhibits proliferation of all 60 cancer cell lines in US National Cancer Institute with average IC_50_ value at 12 nM [32]. Triptolide also reveals a antiproliferative activity on a panel of 12 different cell lines with IC50 values in the 3–70 nM range [33]. All these data suggest that Triptolide is a potent natural anti-tumor compound and has potential for PCa therapy.

Triptolide has been shown to induce apoptosis in many cancer cells *in vitro* or *in vivo*. In this study, we demonstrated that both Triptolide and Celastrol significantly induced apoptosis in the two PCa cell lines tested associated with caspase-3 activation and PARP cleavage. The AR-positive cell line LNCaP seems more sensitive to these two compounds than AR-negative cell line PC-3. Wang et al [34] reported that Triptolide induces apoptosis in cervical adenocarcinoma cells HeLa and pancreatic carcinoma cells PANC-1, associated with activation of caspase-8/9/3 and cleavage of PARP and Bid. Wan et al [35] found that Triptolide induces apoptosis in promyelocytic leukemia cells HL-60 with concomitant DNA fragmentation, S phase cell cycle arrest, mitochondrial cytochrome *c* release and caspases activation. These results indicate that mitochondria-mediated pathway and caspase cascade are involved in Triptolide-induced apoptosis. However, Tan et al [36] reported that potent caspase inhibitor zVAD-fmk causes significant reduction of focal adhesion kinase (FAK) and PARP cleavage, but fails to prevent Triptolide-induced cell death in breast cancer cells MCF-7, suggesting that Triptolide-induced apoptosis is probably mediated through multiple mechanisms and pathways in addition to mitochondria-mediated pathway and caspase cascade. It seems that Triptolide induces apoptosis regardless of p53 status of target cells as both LNCap (p53 intact) and PC-3 (p53 deficient) cells are responsive to Triptolide. The previous reported studies have shown that Triptolide induces apoptosis in these PCa cells without affecting p53 expression [31], and that Triptolide may function differently in various cells with different p53 status [37], pointing the potential efficacy of Triptolide on tumor types with p53 mutation/deletion. Taken together, these data indicate that Triptolide is capable to trigger massive apoptosis in cancer cells through multiple mechanisms and pathways.

Epigenetic alterations play a key role in cancerogenesis and progression [38]. These alterations include not only DNA methylation and covalent histone modification, but also nonhistone protein postmodification like phosphorylation, acetylation, ubiquitylation and SUMOylation, the altered expression of enzymes of these postmodification and the imbalance of these reversible covalent modifications. Since epigenetic aberrations are reversible and can be restored to their normal state, epigenetic therapy to reduce the tumorigenicity promises a novel approach against cancer and is becoming attractive [39]–[41]. For example, successful use of bortezomib, a 26S proteasome inhibitor, for the treatment of multiple myeloma is based on its targeting in ubiquitin/proteasome-mediated pathways [42]–[44]. The therapeutic efficacy of arsenic trioxide in acute promyelocytic leukemia is dependent on the SUMOylation of PML–RARα oncoprotein [21]. These evidences indicate that ubiquitination and SUMOylation play important roles in human cancer progression and could be effective therapy targets. SUMOylation has been linked to tumorigenesis and cancer metastasis [45], [46]. Altered expression of SENPs has been detected in several carcinomas [47]. Balance of SUMOylation and DeSUMOylation is important for cell to maintain normal physiological process. The imbalance perturbs cell fate and initiate cancer development [47]. SENP1, one of SUMO-specific protease which processes the pro-SUMO to mature SUMO and deconjugates the modified proteins is elevated in PCa and plays a regulatory role in PCa development [15]. Although the mechanism underlining the function of overexpressed SENP1 in PCa progression is not clear, SENP1 has been demonstrated to promote many proteins activities playing roles in PCa development. SENP1 reverses the ligand-induced SUMOylation of AR and promotes AR-dependent transcription [48]. SENP1 also promotes AR-mediated transcription via its deSUMOylation on HDAC1 which inhibits the HDAC1 deacetylase activity and transcription suppression ability [17]. SENP1 enhances the c-Jun dependent transcription via deSUMOylation of the CRD1 domain of p300, releasing the *cis* repression of CRD1 domain of p300 [18]. SENP1 is essential for stabilization of hypoxia inducible factor 1 (HIF1α) through its deSUMOylation during the hypoxia-induced process [12]. SENP1 is also essential in endothelial cells as a positive regulator of hypoxia-driven VEGF production and angiogenesis [49]. In the present study, we demonstrated that Triptolide significantly down-regulates SENP1 expression at both mRNA and protein levels and results in an enhanced cellular SUMOylation in PCa cells. The potent efficacy of Triptolide on PCa may be mediated by the down-regulation of SENP1 expression and restoration of cellular SUMOylation level.

We observed that Triptolide suppressed AR expression at mRNA and protein levels and inhibited AR-mediated transcription in AR-positive LNCaP cells. Androgens are critical for the normal prostate development and AR transduces the androgen signals to regulate the network of androgen response genes [50]. On the other hand, androgens are involved in PCa development under pathological situation. The androgen ablation therapy remains a standard treatment for advanced PCa. Most patients show positive response to the treatment but nearly 20% cases develop to hormone-refractory (HR) PCa in 1–2 years. Once the cancer progresses to HRPC stage, the PCa turns more malignant with poor prognostics. Throughout prostate cancer progression including HRPC, AR overexpresses or undergoes mutations [51], [52]. Most identified mutated AR in PCa still retain their transcription activities and are transcriptionally active in response to ligands in addition to androgens. AR co-activators such as SRC-1, SRC-3, TIF-2 [53], [54] which could enhance the transcriptional activity of AR and contribute to the sensitization of AR to low levels of androgen concentrations are also elevated in prostate cancer. These observations suggest that PCa may result from dysregulation of AR activity, pointing that AR could be an important target for PCa therapy. Therefore, it is highly probable that Triptolide inhibits PCa proliferation via suppression of AR expression and consequent break of androgen response network functions. That explains why AR positive LNCaP cells are more sensitive to Triptolide than AR-negative PC-3 cells. Interestingly, SENP1 modulates the AR transcriptional activities via its deSUMOylation on AR and HDAC1 [17]. A positive feedback loop exists between AR and SENP1 in which SENP1 enhances the AR-dependant transcription, while AR potentiates the SENP1 expression by directly binding to the SENP1 promoter [55]. Disruption of this loop significantly blunts proliferation of androgen-dependent PCa cells [55]. Triptolide may disturb the AR and SENP1 loop by suppressing SENP1 and AR expression and consequently inhibiting AR-mediated transcription.

We further demonstrated that Triptolide suppressed c-Jun expression at mRNA and protein levels and c-Jun mediated transcription in PCa cells. c-Jun, in combination with Fos subfamily proteins, forms the early response transcription factor AP-1. AP-1 regulates target genes expression through binding to the AP-1 element in the promoter of these genes that involved in various cellular events including development, cellular proliferation, differentiation, transformation, inflammation, apoptosis and cellular migration [56]. Plenty of evidence indicate that c-Jun is a *bona fide* oncoprotein. c-Jun was found highly expressed in many types cancers such as sarcomas [57], classical Hodgkin's disease and anaplastic large cell lymphoma (ALCL) [58], in which c-Jun promotes cancer cell proliferation and suppresses apoptosis. c-Jun is elevated in PCa associated with cellular proliferation and invasion [25]. In addition, c-Jun acts as an AR co-activator to stimulate AR transactivation by mediating receptor dimerization and subsequent DNA binding [25]. Since the important implication of c-Jun in carcinogenesis and tumor progression, it could be a novel target in cancer treatment. It is highly possible that Triptolide inhibits PCa cell proliferation through down regulation of c-Jun expression and consequent inhibition of c-Jun and AP-1 mediated transcription. The AR-mediated transcription suppression may be partially caused via Triptolide-induced inhibition of c-Jun co-activation. Analysis of c-Jun target genes expression showed the c-Jun mediated transcription was inhibited by Triptolide as a result of c-Jun expression suppression. The Triptolide-induced suppression of SENP1 expression may also involve in this process since SENP1 enhances the c-Jun dependent transcription via deSUMOylation of the CRD1 domain of p300 [18].

In our study, Triptolide shows potent efficacy in suppressing PCa cell proliferation and in inducing apoptosis. We observed that Triptolide down-regulates SENP1, c-Jun and AR expression which are over-expressed in PCa and play a pro-proliferation role. We assume that Triptolide anti-PCa effect could be through down-regulating expression of SENP1, c-Jun and AR. The knockdown and rescue experiments confirm our hypothesis. We found that down-regulation of SENP1, c-Jun or AR expression suppress PCa cell viability, which consist with previous reports that SENP1, c-Jun and AR have pro-proliferation roles in PCa and further suggest that Triptolide anti-PCa effect could be through down-regulation of SENP1, c-Jun and AR expression. Triptolide treatment of PCa cells with silenced SENP1, c-Jun or AR further decreased viable cells while the viability ratios were increased. It is possible that knockdown of SNEP1, c-Jun or AR in PCa cells could dilute targets for Triptolide action. Since Triptolide further decreases cell proliferation in these proteins knockdown PCa cells, which suggests that there could be other genes or proteins involved in Tiptolide function. Over-expression experiments also support above conclusion. Ectopic expression of SENP1, c-Jun or AR increase the viability ratio in Triptolide treated PCa cells, as does the Triptolide binding protein XPB over-expression. Furthermore, co-expression of SENP1, c-Jun and AR induces higher cell viablity ratio. All data indicate that SENP1, c-Jun and AR are potential targets of Triptolide, and down-regulation of these proteins is important for Triptolide anti-PCa toxicity. We notice that individual over-expression or co-expression of SENP1, c-Jun or AR do not suppress completely Triptolide effect. This could result from the possibility that Triptolide suppresses expression of SENP1, c-Jun or AR down-stream target genes or proteins, which disturbs action of over-expressed SENP1, c-Jun and AR. However, we could not exclude that other genes or proteins could be involved in Triptolide induced cell death.

**Figure 8:**
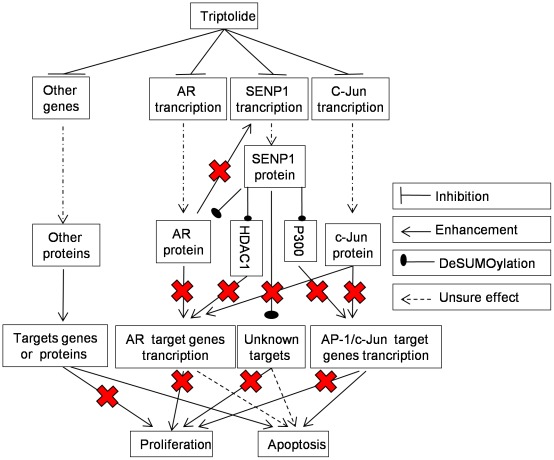
Hypothetic mechanisms of anti-PCa effects of Triptolide. Triptolide suppresses SENP1, AR and c-Jun expression, induces restoration of SUMOylation/deSUMOyaltion balance, inhibitions of the AR and c-Jun mediated transcription, which may all contribute to the anti-PCa effect of Triptolide. Other genes or proteins may involve in mechanism of Triptolide effect (see Discussion for detail).

Triptolide suppresses expression of many genes or proteins in treated cells, including p53, NF-κB, Bcl-2, Mcl-1 [59]. Our study allows adding three new proteins, SENP1, AR and c-Jun, to the list. Understanding the mechanisms underlying these regulations is of major interest. Based on our data and others studies, we propose that Triptolide suppresses PCa progression through multiple pathways (Fig. 8). Triptolide could inhibit SENP1 transcription and decreases SENP1 mRNA and protein levels that are elevated in PCa. Consequently, the cellular SUMOylation is enhanced which could restore the balance of SUMOylation and deSUMOylation, and suppress the functions of some key proteins involved in PCa progression. For example, suppression of SENP1 expression and decrease activity of deSUMOylation on AR and HDAC1 in PCa enhance AR SUMOylation and deacetylation by HDAC1, leading to inhibition of AR-mediated transcription. c-Jun mediated transcription is inhibited by the *cis* repression of CRD1 domain of p300 which could be released by SENP1-mediated deSUMOylation of CRD1 domain. Triptolide could inhibit directly AR expression, inducing suppression of AR mediated transcription. AR down-regulation also inhibits SENP1 expression, enhancing the cellular SUMOylation activities. Triptolide could inhibit directly c-Jun expression to reduce AP-1 and c-Jun mediated transcription. The non-transcriptional function of c-Jun as co-activator also was suppressed. Furthermore, Triptolide could influence other genes expression or target other proteins to disturb these genes or proteins' abnormal functions in PCa. As a result, inhibition of AR and c-Jun mediated transcription, suppression of other targets functions by SENP1 deSUMOylation and interruption of other important molecular functions contribute to inhibit PCa proliferation and progression, and to facilitate apoptosis.

The reason how Triptolide suppresses many genes and proteins expression remains unknown. It's reported that Triptolide could bind a 90 kD unknown nuclear protein [60], which is revealed to be a subunit of the transcription factor TFIIH XPB [61]. Triptolide covalently binds to XPB and inhibits its DNA-dependent ATPase activity, leading to the inhibition of RNA polymerase II–mediated transcription. Triptolide was also found to induce phosphorylation of Rpb1, another subunit of RNA polymerase II, and subsequent proteasome-dependent degradation, which results in a global transcription inhibition [62]. Both XPB and Rpb1 as targets of Triptolide may account for its suppression effect on expression of many genes and its high efficacy on cancer cells. Further studies are required to determine whether Triptolide down-regulates SENP1, c-Jun and AR through targeting XPB and Rpb1.

In summary, our study showed that Triptolide, an active component extracted from Chinese medicinal herb, is an effective agent against prostate cancer. Its anti-tumor activity may be attributed to mechanisms involving down-regulation of SENP1 that restores SUMOylation and deSUMOyaltion balance and negative regulation of AR and c-Jun expression that inhibits the AR and c-Jun mediated transcription in PCa.

## Materials and Methods

### Ethics statement

This study was approved by The Ethics Committee for Animal Experiments of the Fourth Military Medical University (Permit Number: 11004) and the experimental protocol was carried out in strict accordance with the institutional guidelines and the criteria outlined in the “Guide for Care and Use of Laboratory Animals”. All surgery was performed under sodium pentobarbital anesthesia, and all efforts were made to minimize suffering.

### Reagents

Purified Celastrol (>98%) and Triptolide (>98%) were purchased from π-π Technologies, Inc (Shenzhen, China) or Sigma-Aldrich (C0869 & T3652), which were dissolved in DMSO (Wolsen) at a stock concentration of 50 mM and stored at 4°C. Trypan blue was from Sigma-Aldrich. Thiazolyl Blue Tetrazolium Bromide (MTT, M6494), Vybrant Apoptosis Assay Kit #3 (V13242), Trizol Reagent and Lipofectamine™ LTX with Plus™ Reagent were obtained from Invitrogen. High-Capacity cDNA Reverse Transcription Kits was supplied by Applied Biosystems. SYBR® PrimeScript™ RT-PCR Kit II was from TaKaRa (Dalian, China). DharmaFECT® 2 transfection reagent (T200201) was from Dharmacon RNAi Technologies. Antibodies for SENP1 (sc-46634), AR (sc-7305), α-tubulin (sc-5286), β-actin (C4), SUMO-1 (sc-5308) were from Santa Cruz Biotechnology. Another SENP1 antibody (ab58417) was from Abcam. Antibodies for Caspase-3 (#9662), PARP (#9532) and c-Jun (#9165) were from Cell Signaling Technology. Antibodies for Flag (CW0090) and HA (CW0260) tags were from CWBIO (Beijing, China). Rabbit polyclonal SUMO-1 antibody was generated by our laboratory using recombinant SUMO-1according to standard protocol. Secondary antibodies for goat anti-mouse IR Dye 680 (926–32220) and 800 cw (926–32210), donkey anti-goat IR Dye 680 (926–32224) and 800 cw (926–32214), and goat anti-rabbit IR Dye 680 (926–32221) and 800 cw (926–32211) were from LI-COR Biosciences.

### siRNAs and DNA plasmids

ON-TARGET*plus* SMARTpool siRNAs for human SENP1 (L-006357-00), human c-Jun (L-003268-00), human AR (L-003400-00) and Non-targeting control siRNA were purchased from Dharmacon RNAi Technologies. Flag-EGFP, Flag-SENP1, Flag-c-Jun and Flag-XPB were made by standard cloning method. SENP1, c-Jun and XPB coding DNA fragments without ATG were amplified by PCR using the cDNA reverse transcription from LNCaP total mRNA as template. EGFP coding fragment was cloned from pEGFPN1. Cloned fragments were subcloned into BamHI and EcoRI digested pcDNA3 vector with a Flag tag. The sequences of constructs were confirmed by DNA sequencing. The AR expression plasmid Psl2-HA-AR was a gift from Dr. Guangchao Sui (Wake Forest University).

### Cell culture

LNCaP and PC-3 cells, purchased from Institute of Basic Medical Sciences Chinese Academy of Medical Sciences, were maintained in RPMI1640 (GIBCO) supplemented with 10% fetal bovine serum, 100 units/ml of penicillin and streptomycin, and incubated at 37°C with 5% CO_2_.

### Cell proliferation assay and viability assay

LNCaP and PC-3 cells were plated in 24-well plate at a density of 1×10^4^ per well and cultured until attachment, then treated with various doses of Triptolide or Celastrol, using DMSO as negative control and no treatment as blank control. Cells were gently trypsinized and staining with trypan blue dye. The viable cells were counted using cell counting chamber every 24 h for 7 days.

For viability assay, 5×10^3^ LNCaP or 3×10^3^ PC-3 cells per well were plated in 96-well plate and cultured until attachment, then treated with various doses of Triptolide or Celastrol for 48 h, using DMSO as negative control. After adding 100 µl medium containing 10 µl MTT solution (5 mg/ml stock in PBS) per well, the plates were incubated at 37°C with 5% CO_2_ for 4 h. Aspirating the medium, the crystals formed in each well were dissolved in 100 µL DMSO and mix on Shaker for 1 min. The absorbance of each well was measured on a Multilabel counter (PerkinElmer Victor^3^TM 1420) at 595 nm. Each treatment was performed in triplicate and experiments were repeated over 4 times. Data were expressed as percentage of growth inhibition as follows: relative cell viability = (A_595_ (treated)-A_595_ (blank))/(A_595_ (control)-A_595_ (blank)). 50% inhibitory concentration (IC_50_) was calculated from viability assay data with GraphPad Prism 5.04 (GraphPad Software, Inc) using a sigmoidal dose-reponse nonlinear regression analysis.

### Transient transfection and treatment

LNCaP or PC-3 cells were plated in 6- or 12-well plate and cultured until attachment. To knockdown SENP1, c-Jun or AR expression, ON-TARGET*plus* SMARTpool siRNA were transfected into PCa cells using DharmaFECT® 2 transfection reagent according to the manufacturer's instructions. Non-targeting siRNA was used as control. For overexpression, 800 ng each expression plasmid DNA was transfected or co-transfected into PCa cells using Lipofectamine™ LTX with Plus™ Reagent. Empty vector was used as blank control and to keep the total amount of plasmids DNA equal in each well. The plasmids expression irrelevant protein EGFP and Triptolide binding protein XPB were used as negative and positive control, respectively. 48 h after transfection, cells were treated with Triptolide for another 48 h. After stained with trypan blue, viable cells were counted using cellometer Auto T4 automated cell counter.

### Apoptosis analysis

1.5×10^5^ LNCaP or 1×10^5^ PC-3 cells per well were cultured in 6-well plate in which coverslips were plated. After attachment, cells were treated with 1 µM Triptolide or Celastrol for 24 h. Annexin V (AV)/propidium iodide (PI) co-labeling was performed using the Vybrant Apoptosis Assay Kit #3 according to the manufacturer's instructions. The apoptotic cells were analyzed by inverted fluorescence microscopy (Leuca DM IRM) or by flow cytometry (FACSCalibur, Becton Dickinson) using FITC signal detector (FL1) and phycoerythrin emission signal detector (FL2). Data analysis was performed using Expo32 (version 1.2) software.

### Chemiluminescence Immumo-Assay (CLIA)

LNCaP cells were cultured in 6-well plate at a density of 1×10^5^ cells per well and then treated with 1 µM Triptolide or Celastrol for 24 h or 48 h. Aliquots of medium and cell lysate from the treated cells were collected to measure the total PSA level on a chemiluminescence apparatus (Roche Cobas E601) according to protocol of CLIA.

### Real-time PCR

LNCaP or PC-3 cells were cultured in 6-well plate and then treated with various doses of Triptolide or Celastrol for 24 h or with 0.1 µM Triptolide and Celastrol for desired time points. RNA was isolated from treated cell using Trizol Reagent following the manufacturer's instructions. 500 ng total RNA was reverse transcribed to cDNA using High-Capacity cDNA Reverse Transcription Kits. The real-time PCR were performed on the Bio-Rad CFX 96 Real-time PCR system using SYBR® PrimeScript™ RT-PCR Kit II and specific primers (Figure S3). mRNA level of each gene were normalized to β-actin with ΔΔC_T_ method using Bio-Rad CFX Manager V1.1.308.1111 software. The relative mRNA level was calculated by dividing the normalized each gene expression of treated cells with untreated control sample.

### Western blot analysis

LNCaP or PC-3 cells were treated with various doses of Triptolide or Celastrol for 24 h or with 0.1 µM Triptolide or Celastrol for desired times. Cell were trypsinized and washed with cold PBS. Cell pellets were lysed with lysis buffer (50 mM Tris [pH 7.5], 5 mM EDTA, 0.1% NP-40, 300 mM NaCl, with freshly added 0.5 mM phenylmethylsulfonyl fluoride and 1× Roche protease inhibitors cocktail solution) and incubated on ice for 30 minutes with gentle shake or with M-PER protein extraction solution according to the instructions (Thermo Scientific, #78501). Protein samples were separated by 12% SDS-polyacrylamide gel electrophoresis and electrotransferred to nitrocellulose membrane. Protein blots were probed with an appropriate primary antibody and a secondary antibody (IRDye, LI-COR) and then analyzed by quantitative immunoblot using an Odyssey Infrared Imaging System (LI-COR). α-tubulin or β-actin was used as loading control.

### Mouse xenograft assay

5-week-old male nude immunodeficient mice (NCRNU-M) were purchased from Animal Research Center of the Fourth Military Medical University (Xi'an, Shaanxi, China) and maintained in a standard environment. The mice were allowed to acclimatize for at least 1 week before experiments. PC-3 cells (2×10^6^) suspended in 0.1 mL of serum-free RPMI 1640 were inoculated s.c. in the left flank of each mouse. When tumor volume reached 100 mm^3^, mice were divided randomly into control and treatment groups. Mice in treatment group were injected with Triptolide at 0.4 mg/kg daily for 15 days while control mice were injected with vehicle (DMSO). Tumor sizes were measured using calipers and their volumes were calculated using a standard formula: width^2^×length/2, and body weight was measured every three days. The xenograft tissues were collected immediately after the animals were sacrificed and stored at −80°C for further study.

### Statistical analysis

Results were expressed as the mean ± SD. Student's *t* test was applied to evaluate the differences between treated and control groups. For all the tests, the level of significance was set at P<0.05.

## Supporting Information

Figure S1 **Chart for the results from** **Figure 2C** **.** After treatment with Triptolide or Celastrol, LNCaP cells (A) and PC-3 cells (B) were stained with AV/PI and analyzed by flow cytometry. Percentages of viable cells (AV−/PI−), early apoptotic cells (AV+/PI−) and late apoptotic/necrotic cells (AV+/PI+) are presented.
(TIF)

Figure S2 **Triptolide enhanced cellular SUMOylation in PCa cells.** (A) and (B) Triptolide enhanced SUMO-1 heterodimers level in PCa cells. PCa cells were treated with 1 µM Triptolide or Celastrol for 24 h, Cell pellets were lysed in lysis buffer with 20 mM NEM. Western blot were performed using a SUMO-1 monoclonal antibody. (A) Triptolide enhanced SUMO-1 heterodimers levels in PC-3 cells. (B) Triptolide enhanced SUMO-1 heterodimers levels in LNCaP cells. (C) Triptolide decreased SUMO-1 monomer level in LNCaP cells. LNCaP cells were treated with 1 µM Triptolide or Celastrol for 24 h, Cell pellets were lysed in normal lysis buffer. Western blot were performed using a SUMO-1 monoclonal antibody.
(TIF)

Figure S3 **Primers for plasmids construction and Real-time PCR.** Specific primers used for SENP1, c-Jun, XPB and EGFP expression plasmids construction or analysis of SENP1, AR, c-Jun, and AR/c-Jun target genes mRNA levels in Triptolide- or Celastrol-treated PCa cells by Real-time PCR.
(TIF)
