# Supplementary figures and images for: Triptolide Inhibits the Proliferation of Prostate Cancer Cells and Down-Regulates SUMO-Specific Protease 1 Expression
Source: PLoS One. 2012 May 30;7(5):e37693. doi: 10.1371/journal.pone.0037693 (PMC3364364; doi:10.1371/journal.pone.0037693)

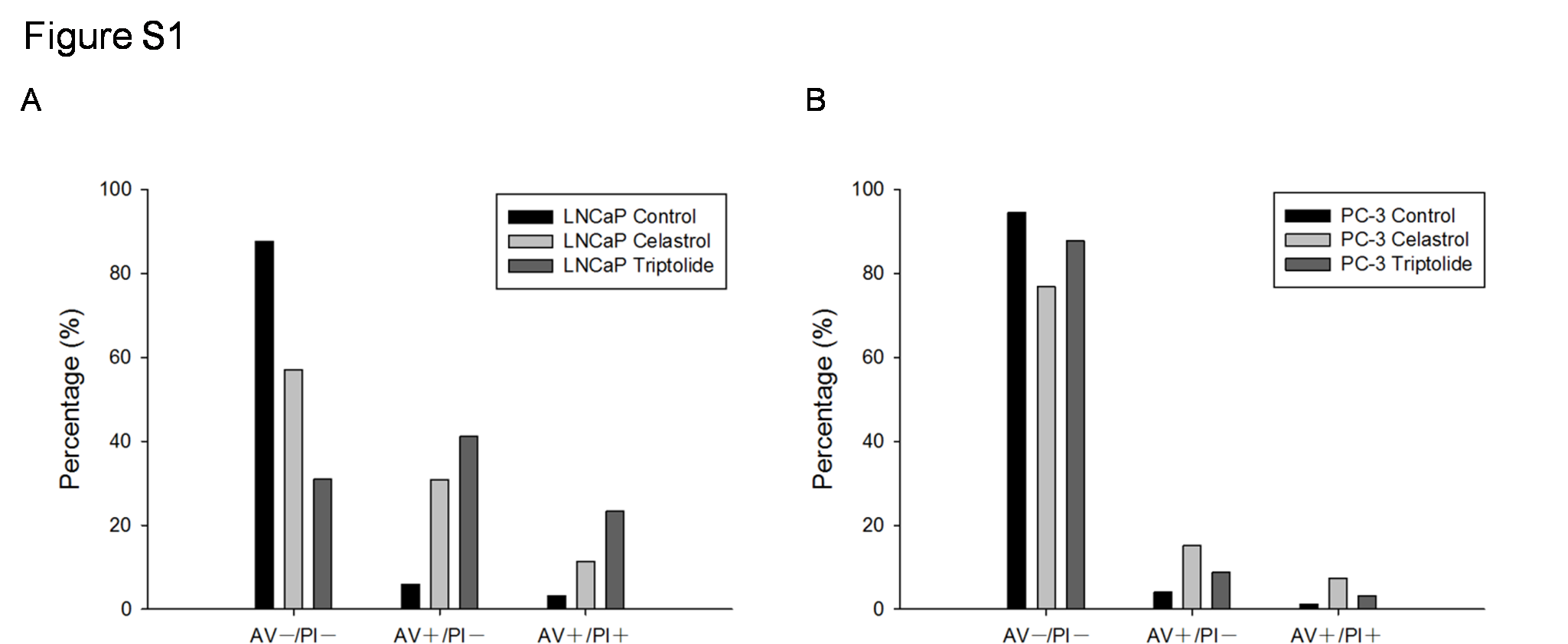

Supplement: Figure S1 — Chart for the results from Figure 2C . After treatment with Triptolide or Celastrol, LNCaP cells (A) and PC-3 cells (B) were stained with AV/PI and analyzed by flow cytometry. Percentages of viable cells (AV−/PI−), early apoptotic cells (AV+/PI−) and late apoptotic/necrotic cells (AV+/PI+) are presented. (TIF) [file pone.0037693.s001.tif]

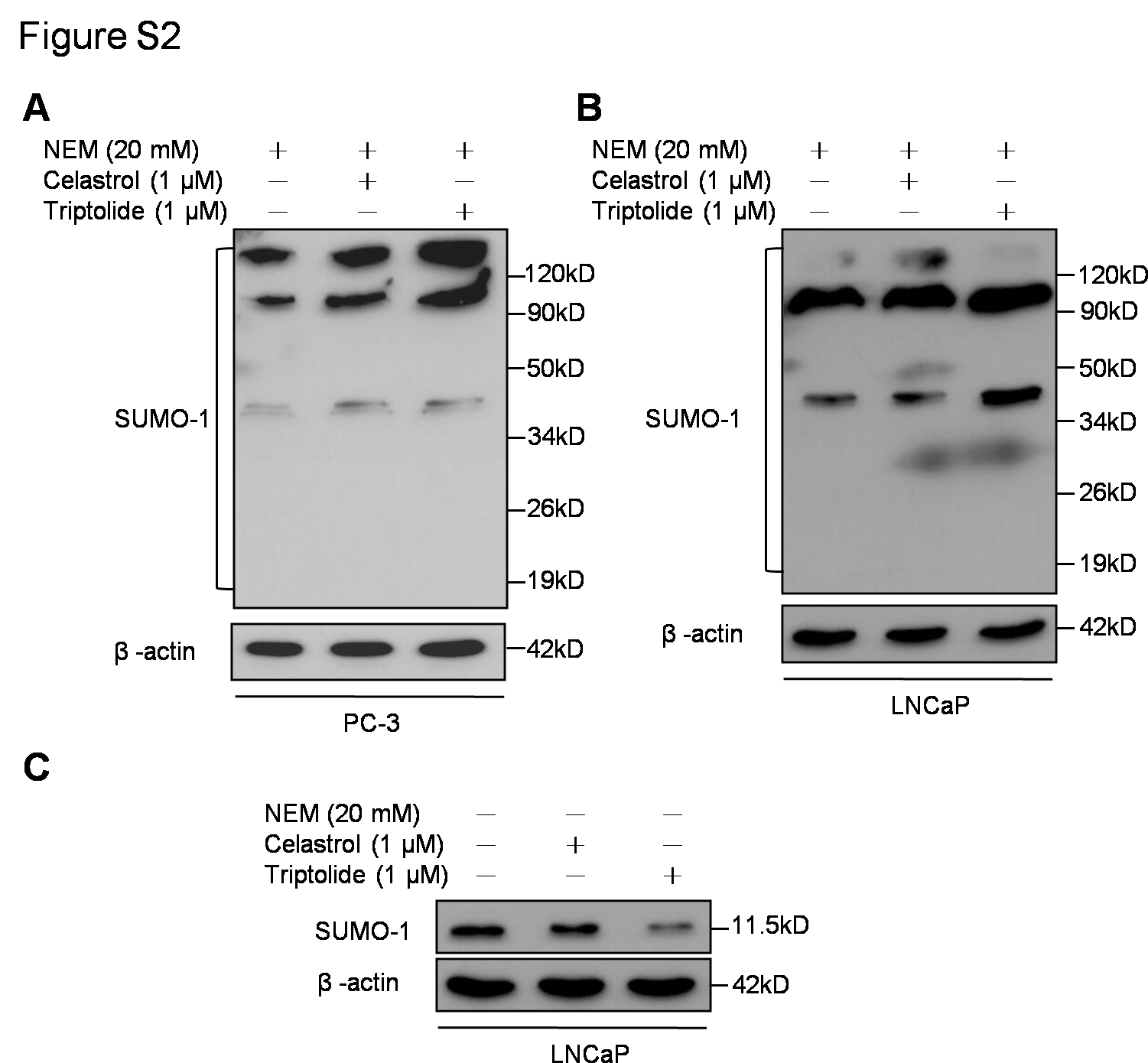

Supplement: Figure S2 — Triptolide enhanced cellular SUMOylation in PCa cells. (A) and (B) Triptolide enhanced SUMO-1 heterodimers level in PCa cells. PCa cells were treated with 1 µM Triptolide or Celastrol for 24 h, Cell pellets were lysed in lysis buffer with 20 mM NEM. Western blot were performed using a SUMO-1 monoclonal antibody. (A) Triptolide enhanced SUMO-1 heterodimers levels in PC-3 cells. (B) Triptolide enhanced SUMO-1 heterodimers levels in LNCaP cells. (C) Triptolide decreased SUMO-1 monomer level in LNCaP cells. LNCaP cells were treated with 1 µM Triptolide or Celastrol for 24 h, Cell pellets were lysed in normal lysis buffer. Western blot were performed using a SUMO-1 monoclonal antibody. (TIF) [file pone.0037693.s002.tif]

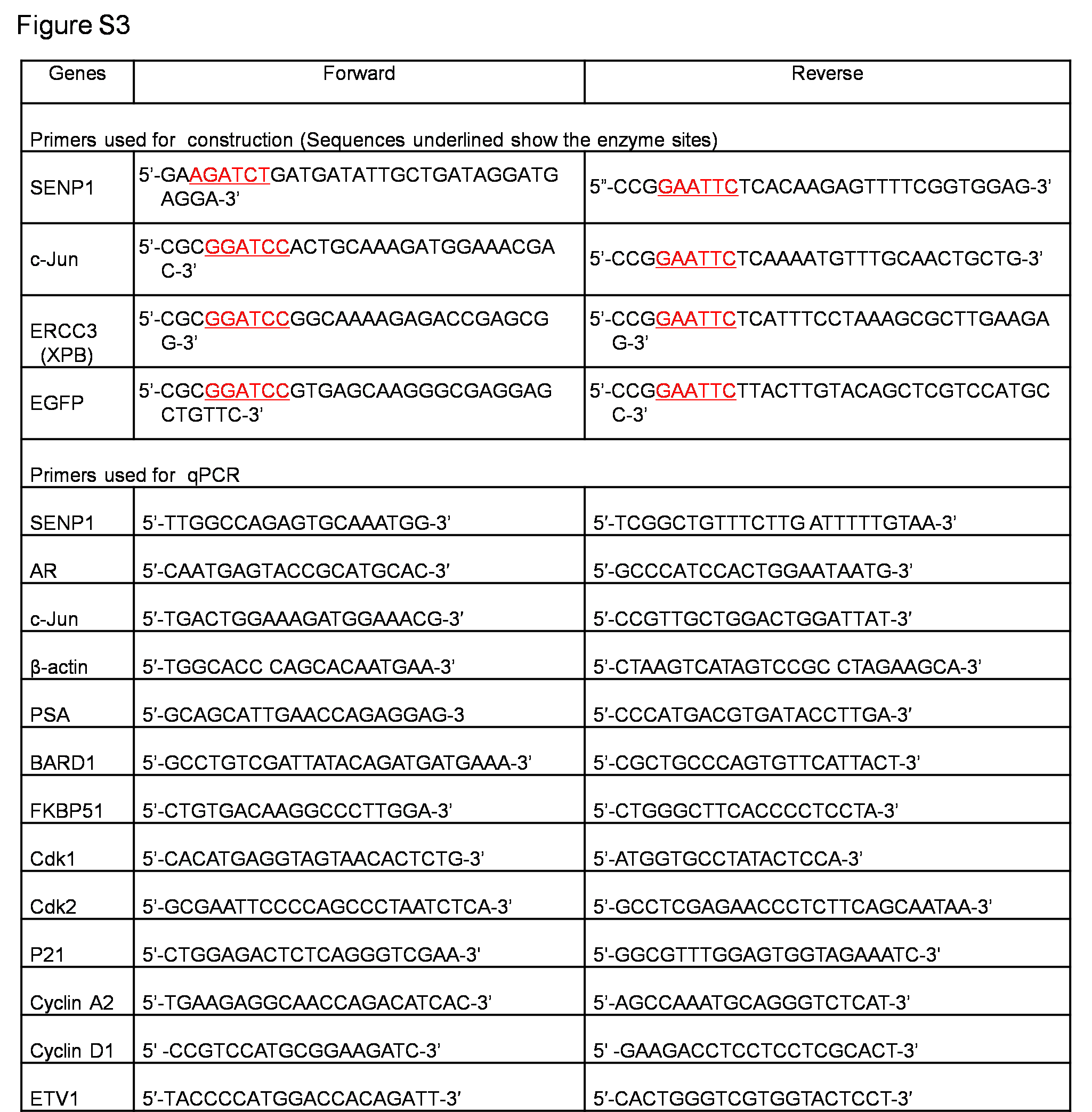

Supplement: Figure S3 — Primers for plasmids construction and Real-time PCR. Specific primers used for SENP1, c-Jun, XPB and EGFP expression plasmids construction or analysis of SENP1, AR, c-Jun, and AR/c-Jun target genes mRNA levels in Triptolide- or Celastrol-treated PCa cells by Real-time PCR. (TIF) [file pone.0037693.s003.tif]
